# Glioma-Induced Seizure in a Neurofibromatosis Type 1 Patient: A Case Report

**DOI:** 10.7759/cureus.19435

**Published:** 2021-11-10

**Authors:** Jonathan Quinonez, Samir Ruxmohan, Sylvia Paesani, Abhinav Patel, Omo Edaki

**Affiliations:** 1 Neurology/Osteopathic Neuromuscular Medicine, Larkin Community Hospital, Miami, USA; 2 Neurology, Larkin Community Hospital, Miami, USA; 3 Family Medicine, Larkin Community Hospital, Miami, USA; 4 Radiology, Northwestern University Feinberg School of Medicine, Chicago, USA; 5 Division of Research and Academic Affairs, Larkin Community Hospital, Miami, USA

**Keywords:** low grade gliomas, all neurology, autosomal dominant genetic disorder, pediatric seizure, neurofibromatosis 1

## Abstract

Neurofibromatosis type 1 (NF-1), also known as Von Recklinghausen's disease, is an autosomal-dominant disease that is characterized by high-frequency mutations leading to multiple benign tumors called neurofibromas and café au lait spots on the skin. Although NF-1 mainly affects the nervous system, it can have multisystem involvement as well, associated with the cardiovascular, orthopedic, gastrointestinal, and dermatologic systems. Psychiatric complications like anxiety, dysthymia, and depression have also been reported in patients with NF-1. The prevalence of this disorder is one in 3,000 births. NF-1 patients have a higher prevalence of seizures compared to the general population.

A 20-year-old male with a diagnosis of NF-1 at the age of three months presented to the emergency room (ER) of a local hospital for the evaluation of an unwitnessed seizure characterized by loss of consciousness and bladder control. MRI of the brain without contrast revealed hyperintensities in the mesial temporal lobe bilaterally, with a hyperintense FLAIR lesion in the splenium of the corpus callosum. The patient exhibited sudden aggression and combativeness while in the ER and also experienced a second seizure, which prompted immediate intubation. A second MRI with contrast confirmed the presence of the lesion. The patient also underwent electroencephalogram (EEG) monitoring later during his hospital stay, the results of which were unremarkable.

This case report discusses an adult male with NF-1 and a tumor of the splenium of the corpus callosum. The displayed imaging suggested a possible etiology for high seizure frequency in patients with NF-1 compared to the general population.

## Introduction

Neurofibromatosis type 1 (NF-1), or Von Recklinghausen's disease, is characterized by the development of multiple benign tumors called neurofibromas that impact the nervous system and skin [1]. The triad of café-au-lait macules, skinfold freckling, and Lisch nodules are distinctive features of this disorder [1]. The underlying defect is the germline mutation in the NF-1 tumor suppressor gene on chromosome 17, which is transmitted as an autosomal-dominant trait. The outcome of this genetic mutation can result in varying presentations in affected individuals. Skeletal abnormalities like scoliosis, tibial pseudarthrosis, and orbital dysplasia as well as peripheral nerve and brain tumors, adversely affect the daily activities of these individuals, thereby impacting their quality of life [1,2,3]. Furthermore, comorbidities that include psychiatric disorders and seizures, when present, can result in a patient’s functional decline. Although NF-1 patients have a higher seizure prevalence compared to the general population, epilepsy is seen in about 4-7% of patients with this disorder [2,3,4]. Infantile spasms and febrile seizures may also be seen in patients with NF-1 [4]. The prevalence of this disorder is one in 3,000 births and the life expectancy will depend on the presence of comorbidities and the quality of life of the affected patient. In this report, we present a rare case of glial-induced seizure in a patient with a history of NF-1.

## Case presentation

The patient was a 20-year-old male with a past medical history of NF-1 who presented to a local hospital for the evaluation of an unwitnessed seizure event. The patient had been born at full term without any complications. He had not exhibited any developmental delays or loss of milestones. He had been diagnosed with NF-1 at the age of three months when he presented with multiple café-au-lait spots; he also had a family history of NF-1. Since his diagnosis, the patient had been relatively asymptomatic except for hydrocele surgery at the age of three. He had later exhibited multiple small, non-painful neurofibromas over the trunk and extremities as well as a left inner thigh plexiform neurofibroma. A past MRI of the head and orbit without contrast demonstrated the following findings:

· No evidence of an optic pathway tumor

· Evidence of signal abnormalities involving the basal ganglia, brainstem, cerebellum, mesial temporal region, and thalamus

· Spongiotic and vacuolar changes of myelin within the above regions

Before the current presentation, the patient had experienced an unwitnessed seizure event. He had been later found by his mother, and the patient had exhibited increased body tone, loss of bladder tone, as well as loss of consciousness. It was unknown if the patient had exhibited any tonic-clonic jerking. His seizure episode had lasted about one and a half to two minutes. He had already had two previous episodes two years ago, which had occurred back-to-back. With his current episode, the post-ictal period had lasted for approximately 40 minutes and was described as “an episode of fatigue.”

After admission, a physical examination was unremarkable except for numerous café-au-lait spots noted on his skin, multiple small neurofibromas of less than 1 cm over the back and extremities, and a large discolored plexiform neurofibroma with a size of 10 x 10 cm over the left medial inner thigh. There was no tenderness or induration to the large neurofibroma. Otherwise, he was alert and oriented to person, place, and time and cognitively intact.

An initial MRI of the brain demonstrated hyperintensities over the left mesial temporal lobe and a hyperintensity FLAIR near the left splenium of the corpus callosum. The patient became combative, and hence only an MRI of the brain without contrast was done, and he later experienced another seizure event. He was intubated and underwent a repeat MRI of the brain with contrast, which showed a faint enhancement of the splenial lesion. 

**Figure 1:**
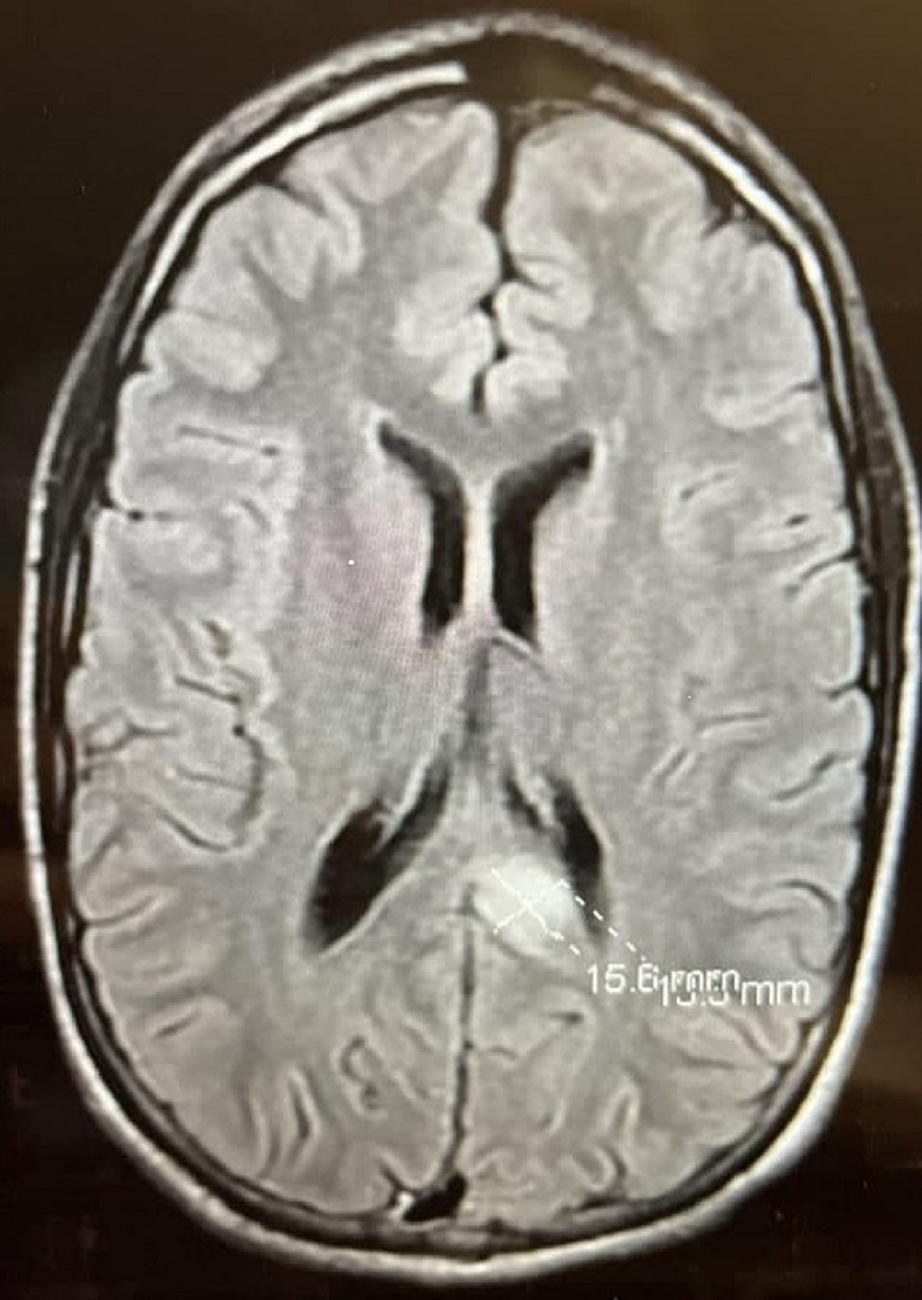
MRI brain with contrast showing enhancement of the splenial lesion MRI: magnetic resonance imaging

Due to his second seizure episode, he was admitted for further observation. Further evaluation of the patient’s recent MRI of the brain demonstrated no clear etiology of the left splenial lesion measuring 1.5 cm x 1.6 cm as shown in Figure 1. Other important findings included focal areas of high signal intensity seen in the cerebellar hemispheres, brainstem, thalamus, and basal ganglia as well as hippocampal hyperintensities. As a result, levetiracetam was started, and the patient was discharged home afterward. He was later admitted again for an overnight video electroencephalogram (EEG) for further evaluation, which demonstrated no abnormal events. A repeat read of his most recent MRI of the brain with contrast confirmed the presence of a left splenial lesion. Given his clinical history, radiology and clinical findings supported a differential diagnosis of a neoplasm (low-grade glioma) versus post-seizure hyperemia versus left medial temporal lobe sclerosis. A biopsy of this lesion could not be performed due to the high-risk nature of the procedure given the size of the lesion and its location.

## Discussion

The underlying etiology of seizures in patients with NF-1 is unknown but is thought to be due to the presence of neurofibromatosis bright objects (NBOs) [5,6]. NBOs are identified on a brain MRI due to T2 signal changes in the subcortical region and may suggest an increased risk of seizures when present in high numbers [5,6]. Our patient exhibited an increased body tone, loss of consciousness, and a loss of bladder control lasting about two to three minutes, which are classically associated with a tonic-clonic seizure, despite the absence of jerking in extremities and being an unwitnessed event [7]. The treatment for seizure in patients with NF-1 depends on the type of seizure present. Our patient was given levetiracetam for seizure prevention soon after his seizure spontaneously resolved.

Patients with NF-1 are predisposed to brain tumor development. Around 15-20% of all children with NF-1 develop gliomas, particularly low-grade optic pathway gliomas (LGOPG) [8,9]. LGOPGs can be asymptomatic or unprogressive; over time, however, it can lead to eye proptosis, vision loss, and precocious puberty in about 50% of pediatric patients with females affected three to five times more than males [8]. Caucasian children have been found to have a higher prevalence of LGOPG than other races [8]. Unlike LGOPGs, brainstem gliomas (BSG) affect fewer than 10% of all patients with NF-1, and this type of tumor is not influenced by gender [9]. BSGs typically involve the brainstem and medulla and can result in obstructive hydrocephalus due to aqueduct stenosis [9]. Both BSGs and LGOPGs are diagnosed around the age of seven and are low-grade tumors seen in patients with NF-1. An important consideration is that gliomas can occur in other areas of the central nervous system (basal ganglia, thalamus, spinal cord). If a glioma arises in the thalamus, then this results in a poor prognosis as this is often a high-grade glioma (anaplastic astrocytoma and glioblastoma) [10]. Concerning the above patient, a low-grade glioma was identified based on the appearance of a left splenic lesion with areas of high-frequency intensity in the cerebral hemisphere as identified on an MRI. A biopsy was not performed due to the high-risk nature of the procedure given the lesion's location, but a low-grade glioma most likely caused the patient’s presenting symptoms.

Patients with NF-1 also exhibit an increased rate of anxiety and depression compared to the general population [11,12]. These symptoms in NF-1 are similar to symptoms seen in coronary artery disease, diabetes mellitus, or multiple sclerosis, suggesting that psychiatric presentations may be a consequence of neuropathological changes rather than external factors [12]. Anxiety, which has a multifactorial etiology, can also arise due to neurodevelopmental changes in patients with NF-1. Our patient exhibited sudden agitation while in the emergency department, due to which an MRI with contrast could not be done.

## Conclusions

Patients with NF-1 have a higher rate of seizure occurrence than the general public. MRI imaging characteristics can help discern a possible rationale for this higher seizure frequency in NF-1 patients. Clinicians should be aware of NBOs to help discern a possible cause of seizures in NF-1 patients.
